# Porous COS@SiO_2_ Nanocomposites Ameliorate Severe Acute Pancreatitis and Associated Lung Injury by Regulating the Nrf2 Signaling Pathway in Mice

**DOI:** 10.3389/fchem.2020.00720

**Published:** 2020-10-07

**Authors:** Qixiang Mei, Guoying Deng, Zehua Huang, Yue Yin, Chunlin Li, Junhui Hu, Yang Fu, Xingpeng Wang, Yue Zeng

**Affiliations:** ^1^Shanghai Key Laboratory of Pancreatic Disease, Shanghai JiaoTong University School of Medicine, Shanghai, China; ^2^Department of Gastroenterology, Shanghai General Hospital, Shanghai Jiao Tong University School of Medicine, Shanghai, China; ^3^Trauma Center, Shanghai General Hospital, Shanghai Jiao Tong University School of Medicine, Shanghai, China; ^4^Shanghai General Hospital, Shanghai Jiao Tong University School of Medicine, Shanghai, China

**Keywords:** severe acute pancreatitis, lung injury, oxidative stress, porous COS@SiO2 nanocomposites, Nrf2 signaling pathway

## Abstract

Severe acute pancreatitis (SAP) is associated with high rates of mortality and morbidity. Chitosan oligosaccharides (COSs) are agents with antioxidant properties. We developed porous COS@SiO_2_ nanocomposites to study the protective effects and mechanisms of COS nanomedicine for the treatment of acute pancreatitis. Porous COS@SiO_2_ nanocomposites released COSs slowly under pH control, enabling sustained release and maintaining the drug at a higher concentration. This study aimed to determine whether porous COS@SiO_2_ nanocomposites ameliorate SAP and associated lung injury. The SAP model was established in male C57BL/6 mice by intraperitoneal injection of caerulein. The expression levels of myeloperoxidase, malondialdehyde, superoxide dismutase, nuclear factor-kappa B (NF-κB), the NOD-like receptor protein 3 (NLRP3) inflammasome, nuclear factor E2-related factor 2 (Nrf2), and inflammatory cytokines were detected, and a histological analysis of mouse pancreatic and lung tissues was performed. In the SAP groups, systemic inflammation and oxidative stress occurred, and pathological damage to the pancreas and lung was obvious. Combined with porous COS@SiO_2_ nanocomposites before treatment, the systemic inflammatory response was obviously reduced, as were oxidative stress indicators in targeted tissues. It was found that Nrf2 was significantly activated in the COS@SiO_2_ treatment group, and the expressions of NF-κB and the NLRP3 inflammasome were notably decreased. In addition, this protective effect was significantly weakened when Nrf2 signaling was inhibited by ML385. This demonstrated that porous COS@SiO_2_ nanocomposites activate the Nrf2 signaling pathway to inhibit oxidative stress and reduce the expression of NF-κB and the NLRP3 inflammasome and the release of inflammatory factors, thus blocking the systemic inflammatory response and ultimately ameliorating SAP and associated lung injury.

## Introduction

Acute pancreatitis (AP) is a common acute abdomen presentation in the clinic with increasing morbidity in recent years. The severity of AP can be described as mild, moderate, or severe according to the local injury to the pancreas and systemic injury to other organs (Banks et al., 2013). Severe acute pancreatitis (SAP) is a serious illness with rapid onset and a high fatality rate. It is characterized by the systemic inflammatory response syndrome, the multiple organ dysfunction syndrome, sepsis, and other complications (Bi et al., 2015).

Acute lung injury (ALI) is one of the most serious and earliest complications of SAP. ALI is described as an important risk factor for death in the early stages of SAP with a high mortality rate in the range of 30–40% (Zhou et al., 2010).

Recent studies have shown that oxidative stress is one of the pathophysiological mechanisms for AP (Pérez et al., 2015; Xie et al., 2017). During the pathogenesis of AP, the injured pancreatic cells and the activated immune cells release abundant reactive oxygen species (ROS), which can lead to an imbalance between the oxidation system and the antioxidant system (Hackert and Werner, 2011; Esrefoglu, 2012). This imbalance results in tissue damage, including to the pancreas and other organs such as the lungs, liver, and so on (Fukumoto et al., 2013). Overall antioxidative stress therapy seems to be a key to ameliorating SAP and its associated lung injury.

Chitosan oligosaccharides (COSs) are the degraded product of chitin and chitosan and consist of glucosamine linked by β-1,4-glycosidic bonds. COSs are made from chitosan or chitin derived from shrimp and crab shells by chemical or enzymatic hydrolysis (Muanprasat et al., 2015). COSs are popular because of the variety of their functional biological activities; for example, they have antioxidant, anti-inflammatory, antibacterial, and immunomodulatory properties (Yuan et al., 2019). Several studies have shown that COSs can inhibit oxidative damage to the liver and lung (Junyuan et al., 2018; Tao et al., 2019). Because of their low molecular weight and high solubility, COSs degrade rapidly *in vivo*.

Porous silica nanoparticles with a specific structure and specific surface properties have good biocompatibility and are often used as inorganic non-metallic nanomaterials in biological applications (Cao et al., 2016b; Su et al., 2019). Porous silica nanoparticles are also often used in drug delivery systems (Liu et al., 2015; Su et al., 2019; Zhu et al., 2019). They can release the drug slowly by means of pH control, achieving sustained release and maintaining a higher concentration of the drug (Cao et al., 2016a; Zou et al., 2016; Zhang et al., 2017). In addition, porous silica nanoparticles have the advantage of targeted drug delivery, so that the drug can reach a higher concentration in the target tissue (Rosenholm et al., 2009; Shahbazi et al., 2012; Giret et al., 2015).

We used porous silica nanoparticles loaded with COSs. The porous structure gives COS@SiO_2_ nanospheres the potential to be multifunctional. Thus, in this study, we used an animal model to evaluate the effects of porous COS@SiO_2_ nanocomposites on SAP and associated lung injury.

## Materials and Methods

### Synthesis of Porous COS@SiO_2_ Nanocomposites

COSs (molecular weight <3,000) were purchased from Shanghai Yuanye Bio-technology Co., Ltd. Cetyltrimethylammonium bromide (CTAB), tetraethyl orthosilicate (TEOS), and absolute ethyl alcohol were obtained from Aladdin Reagent Co., Ltd.

The porous silica nanospheres were prepared according to a previously described method (Yang et al., 2012) with some modification. A total of 0.3 g of CTAB was dissolved in 60 ml deionized water, and 9 ml ethylene glycol and 4 ml ammonium hydroxide were added. The mixture was heated to 50°C, and then 4 ml of TEOS was added dropwise with stirring. After reaction for 3 h, the white products were collected by centrifugation and were washed with water. Then the white products were dried, and calcined at 550°C for 3 h in a muffle furnace to remove the surfactant. Finally, the porous silica nanospheres were obtained for further use.

A total of 20 mg of the porous SiO_2_ nanospheres described above were added to the COS solution (5 mg/mL) with stirring. The COSs were loaded into the porous SiO_2_ nanospheres through the porous structure. After stirring at room temperature for 12 h, the COS-loaded porous silica nanospheres (COS@SiO_2_) were obtained by centrifugation and washed with water. Then, 5 mg COS@SiO_2_ was dispersed in 5 ml phosphate-buffered saline with stirring. After 12 h, the supernatant was collected by centrifugation to measure the released COSs.

### Reagents

Caerulein (HY-A0190) and ML385 (HY-100523) were obtained from MedChem Express (Shanghai, China). Lipopolysaccharide (LPS; L2880) was purchased from Sigma-Aldrich (MO, USA). The antibodies against phospho-nuclear factor-kappa B (p-NF-κB) (3033s), NOD-like receptor protein 3 (NLRP3) (1510s), cleaved interleukin 1β (IL-1β) (52718s), caspase-1 p20 (52718s), and histone H3 (14269s) were purchased from Cell Signaling Technology (MA, USA). The antibodies against nuclear factor E2-related factor 2 (Nrf2) (A0674) and β-tubulin (AC008) were purchased from Abclonal (Wuhan, China).

### Animals and Treatments

All animal experiments were conducted according to the guidelines of the Animal Care and Use Committee of Shanghai Jiaotong University and were approved by the Animal Ethics Committee of Shanghai Jiaotong University School of Medicine (SYXK 2013-0050, Shanghai, China.). Male C57BL/6 mice (6–8 weeks old, 20–22 g) were purchased from Shanghai SLAC Laboratory Animal Co., Ltd (China) and maintained in standard cages in a humidity-controlled room with an ambient temperature of 23 ± 2°C and a 12 h light–dark cycle.

The mice were randomly divided into four groups, as follows.

1. Caerulein-induced AP (SAP) group: mice were injected intraperitoneally with caerulein (100 μg/kg, with a 1 h interval between injections) 10 times; LPS (5 mg/kg) was administered by intraperitoneal injection immediately after the last injection of caerulein (Lerch and Gorelick, 2013).
2. Control (CON) group: mice were injected with normal saline instead of caerulein.
3. COS+SAP (SAP) group: mice were first injected with COS (20 mg/kg); after half an hour they were continually injected with caerulein and LPS to induce SAP.
4. COS@SiO_2_+SAP (COS@SiO_2_) group: mice were injected with a corresponding dose of COS@SiO_2_ and then SAP was induced.

In addition, we used another SAP model of mice: L-arginine-induced AP, which is also non-invasive and provides a rapid induction of SAP. To induce AP in mice with L-arginine, the mice were injected intraperitoneally with 8% L-arginine (dose 4.5g/kg) twice, with an interval of 1 h (Lerch and Gorelick, 2013). The grouping of mice with L-arginine-induced AP is the same as for mice with caerulein-induced AP.

Next, we used ML385, an Nrf2 inhibitor, to treat mice in order to explore the specific mechanism of action. In this group (ML385+COS@SiO_2_+SAP group), mice were injected intraperitoneally with ML385 (10 mg/kg); after half an hour, they were continually treated as for the COS@SiO_2_+SAP group. In this group, we only used the caerulein-induced AP model.

Mice were sacrificed at the time points indicated in Figure 2A after being anesthetized with chloral hydrate. Blood samples were taken from the eyeball for each mouse, and the pancreatic tissue and lung tissue were collected and fixed in 4% paraformaldehyde for histological examination or stored at −80°C after freezing in liquid nitrogen for other purposes.

### Histological Examination

After sacrifice, pancreatic tissue and lung tissue were harvested, rinsed, and fixed in 4% paraformaldehyde at 4°C overnight. The tissues were then processed with sequential clearing, rehydrating, and dehydrating steps, and embedded in paraffin blocks. Samples were sectioned into 4 μm slices and subjected to standard hematoxylin and eosin (H&E) staining. H&E images were captured using a light microscope (Leica, Germany) at a magnification of ×100 or ×200. Pancreatic histopathology scores were evaluated by the Schmidt criteria (Schmidt et al., 1992) for pancreatic tissue by edema, hemorrhage, necrosis, and inflammatory infiltration. Lung histopathology scores were evaluated by alveolar neutrophils, interstitial neutrophils, hyaline membranes, proteinaceous debris, and alveolar septal thickening, as described previously (Matute-Bello et al., 2011). The results of all experiments were analyzed blind by two pathologists.

### Pancreas and Lung Wet-to-Dry Weight Ratio

The pancreas and lungs were sucked dry^1^ and weighed, and then baked in an oven at 80°C for 48 h until a constant weight was obtained as the dry weight. To evaluate tissue edema, the ratio of wet lung weight to dry lung weight was calculated.

### Serum Amylase and Lipase Assay

The serum activity of amylase and lipase was measured by enzymatic kinetic chemistry using commercial kits according to the manufacturer's instructions (Roche/Hitachi modular analysis system; Roche, Berlin, Germany).

### Enzyme-Linked Immunosorbent Assay

The blood samples that were collected from the mouse eyeballs were centrifuged at 3,000 rpm for 20 min at 4°C, and the upper serum was frozen at −80°C. Inflammatory cytokines, including IL-6, IL-1β, IL-10, and tumor necrosis factor-α (TNFα), were detected by Luminex Screening Human Magnetic Assay (R&D Systems, Inc., Minneapolis, MN, USA), which was performed according to the manufacturer's instructions. The serum samples were diluted 1:2 using PH7.4 PBS for the assay, and 50 μl of the diluted samples was added to each well. Fluorescence intensity was measured using Microplate Reader and analyzed using software (xponent 3.1; Luminex, Austin, TX, USA).

The pancreatic and lung tissues (30–50 mg) were homogenized and lysed in a RIPA lysis buffer with protease inhibitors, followed by centrifugation (12,000 *g*, 20 min). The samples were used to detect the oxidative stress-related indices [myeloperoxidase (MPO), malondialdehyde (MDA) and superoxide dismutase (SOD)] by enzyme-linked immunosorbent assay (ELISA) (Multiscience Lianke Biotech, Co., Ltd, China).

### Western Blot Analysis

The pancreatic and lung tissues (30–50 mg) were homogenized in ice-cold RIPA buffer (Beyotime, China) containing protease inhibitor (Beyotime, China). Protein samples (10 μg) were separated by 10% sodium dodecyl sulfate–polyacrylamide gel electrophoresis and transferred onto polyvinylidene difluoride membranes (Millipore, USA) by wet electroblotting. The membranes were blocked with 5% bovine serum albumin (Yeasen, China) in Tris-buffered saline containing 0.1% Tween 20 for 1 h at room temperature, incubated with Nrf2 (1:1,000), p-NF-κB (1:1,000), NLRP3 (1:1,000), caspase-1 p20 (1:1,000), cleaved IL-1β (1:1,000), histone H3 (1:1,000), and β-tubulin (1:1,000) antibodies overnight at 4°C, and subsequently incubated with horseradish peroxidase-conjugated anti-rabbit secondary antibodies (1:4,000). Proteins were eventually visualized by utilization of an enhanced chemiluminescent (ECL) kit (Yeasen, China) and normalized to the expression level of β-tubulin.

### Statistics

All measurement data are expressed as the mean ± standard deviation, and statistical analysis was performed using the GraphPad Prism 7.0 software. The *t*-test was used for the normal distribution and comparison between the two groups. One-way analysis of variance (ANOVA) was used for comparisons between the three groups, and the Kruskal–Wallis test was used for data that did not satisfy a normal distribution. *p* < 0.05 indicates that the difference was statistically significant.

## Results

### Characterization of Porous COS@SiO_2_ Nanocomposites

**Figure 1:**
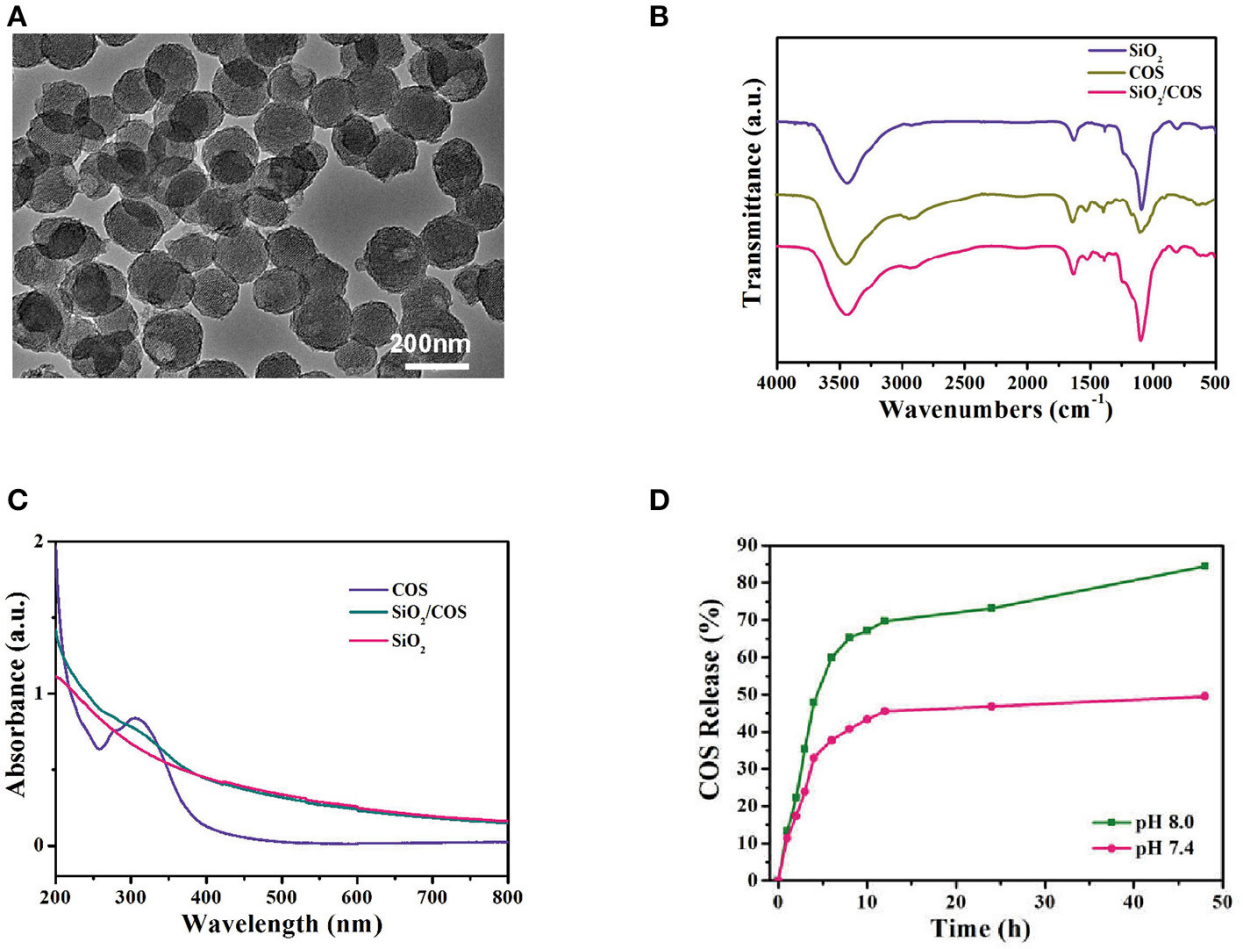
The characterization of porous COS@SiO_2_ nanocomposites. **(A)** Transmission electron microscopy images of COS@SiO_2_. **(B)** Fourier transform infrared spectra and **(C)** ultraviolet**–**near-infrared absorption spectra of SiO_2_, COS, and COS@SiO_2_. **(D)** The cumulative release of chitosan oligosaccharides (COSs) from COS@SiO_2_ at pH 7.4 and pH 8.0.

The porous SiO_2_ was first synthesized using a sol–gel method (Yang et al., 2012). The size of the SiO_2_ nanoparticles was ~110 nm and they exhibited an obvious porous structure (Figure 1A), which could be used for loading COSs. When SiO_2_ was mixed with the COS solution, the COSs were loaded into the SiO_2_ spheres because of their abundant pores. After loading the COSs, COS@SiO_2_ appeared as COS absorption peaks (Figure 1B), indicating that the COSs had been incorporated into the porous SiO_2_. Compared with the smooth curve of SiO_2_, the COS@SiO_2_ curve showed an obvious peak around 305 nm in the ultraviolet–near-infrared absorption spectrum, which further proved successful loading of COS (Figure 1C). The highest loading efficiency of COSs reached 10.2%. COS release was studied at pH 7.4 and pH 8.0. The COS release was much more rapid and the release rate was higher in an alkaline environment, which could be attributed to the enhanced aqueous solubility of COSs (Figure 1D). In the model of AP induced by caerulein, caerulein can promote the contraction of the gallbladder, rupture of the pancreatic duct and acinar cells, and then the release of pancreatin and pancreatic juice into the pancreatic interstitium (Kaiser et al., 1995; Sata et al., 1996). The pancreatic juice is alkaline, and the release rate and amount of COSs increase in an alkaline environment, so COS@SiO_2_ can reach a higher concentration in the local pancreas during AP. Porous silica nanoparticles give COSs the ability to collect in the pancreas and then be released in a high concentration. Hypothetical mode of action of COS was shown in supplement information (Supplementary Figure 1).

### Porous COS@SiO_2_ Nanocomposites Alleviated Pancreatic and Lung Injuries in Mice With Caerulein-Induced AP

**Figure 2:**
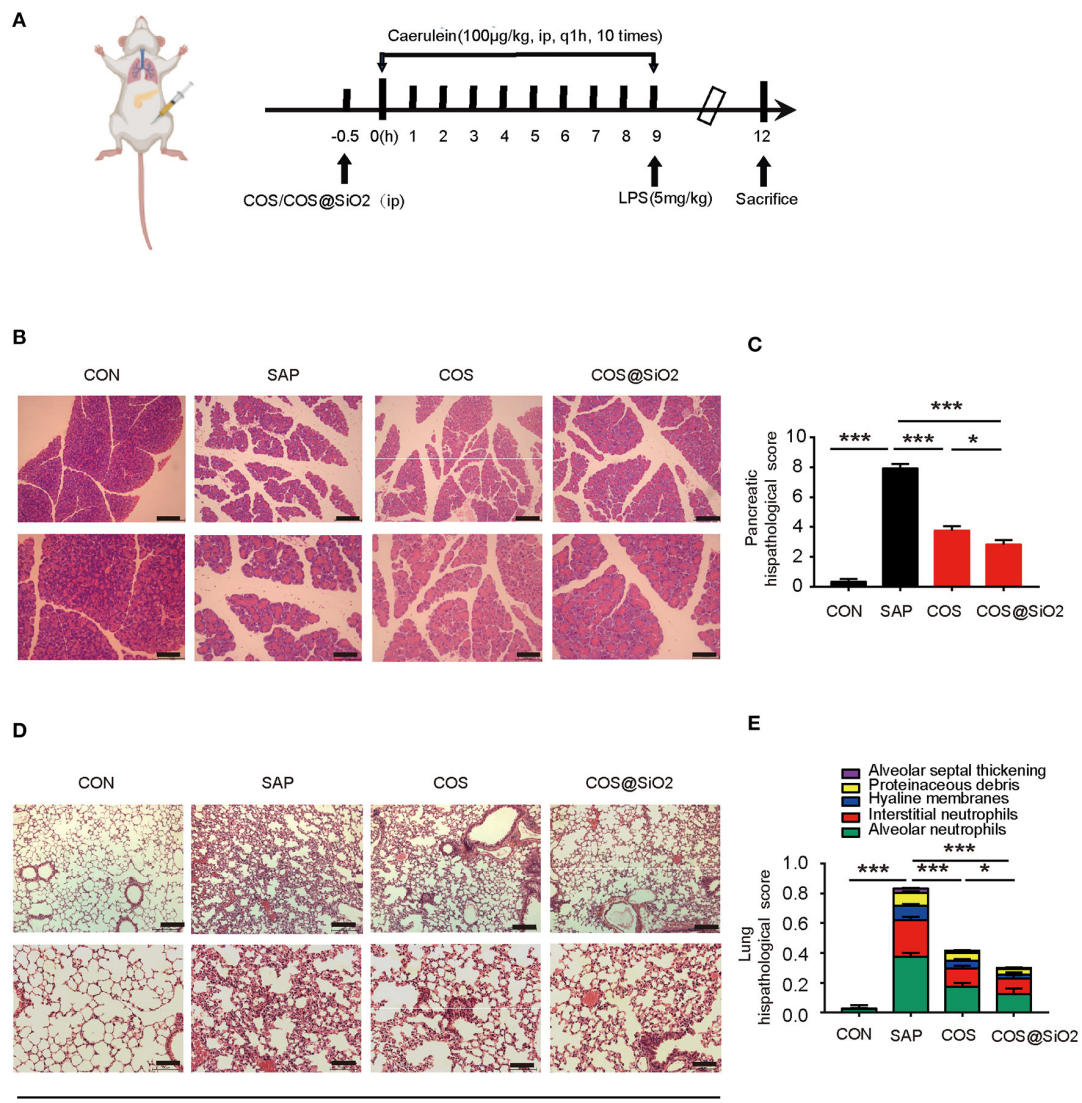
Porous COS@SiO_2_ nanocomposites alleviated pancreatic and lung histopathology injuries in mice with caerulein-induced acute pancreatitis (AP). **(A)** The time axis of AP model building and drug intervention. **(B,C)** Pancreatic samples from the four groups of mice were stained with H&E. Representative images of the pancreas are shown. Original magnification ×100 (upper figures) or ×200 (lower figures). The pancreatic histopathology scores were evaluated by Schmidt criteria for pancreatic tissue by edema, hemorrhage, necrosis, and inflammatory infiltration. **(D,E)** Lung histopathology scores were evaluated by alveolar neutrophils, interstitial neutrophils, hyaline membranes, proteinaceous debris, and alveolar septal thickening. The data are provided as the mean ± SEM (*n* = 6 per group). *p < 0.05; ***p < 0.001.

The results showed that in the control group and the COS@SiO_2_-treated group (Supplementary Figure 2) there was no significant change in pancreatic and lung tissue. There was slight intralobular septal expansion, but no bleeding, necrosis, inflammatory cell infiltration, or other pathological changes. Compared with the control group, there was no obvious pathological damage to the pancreas or lung and no distinct change in serum amylase in the experimental group. The SAP model was successfully established by intraperitoneal injection of caerulein and LPS in mice (Figure 2A). Some pathological changes in the pancreatic tissue were observed, such as obvious edema (intralobular, interacinar, widened intercellular space), hemorrhage, acinar necrosis, and inflammatory infiltration. Compared with the SAP group, pathological injury to the pancreas in the COS and COS@SiO_2_ groups was significantly reduced, and the pathological score was significantly lower (*p* < 0.001). Porous COS@SiO_2_ nanocomposites displayed a higher capacity to attenuate injury to the pancreas induced by caerulein (Figures 2B,C).

We also observed the lungs under light microscope. We found that the lung tissue structure of the control group was clear, the alveolar wall was not significantly thickened, and there was no obvious inflammatory cell infiltration in the interstitium and alveoli.

In the SAP group, the alveolar wall was obviously thickened, the alveolar space was obviously widened, the alveolar cavities and interstitium were infiltrated by a large number of neutrophil inflammatory cells, and the alveoli were filled with protein fragments and had formed a hyaline membrane. Compared with the SAP group, pathological injury to the lung tissue in the COS and COS@SiO_2_ treatment groups was significantly reduced, and the pathological score was significantly different (*p* < 0.001). The pathological injury in the COS@SiO_2_ group was further reduced compared with the COS group, and the pathological score was statistically different (*p* < 0.05; Figures 2D,E). Hence, these results show that porous COS@SiO_2_ nanocomposites can reduce lung histopathology injuries in caerulein-induced AP in mice.

**Figure 3:**
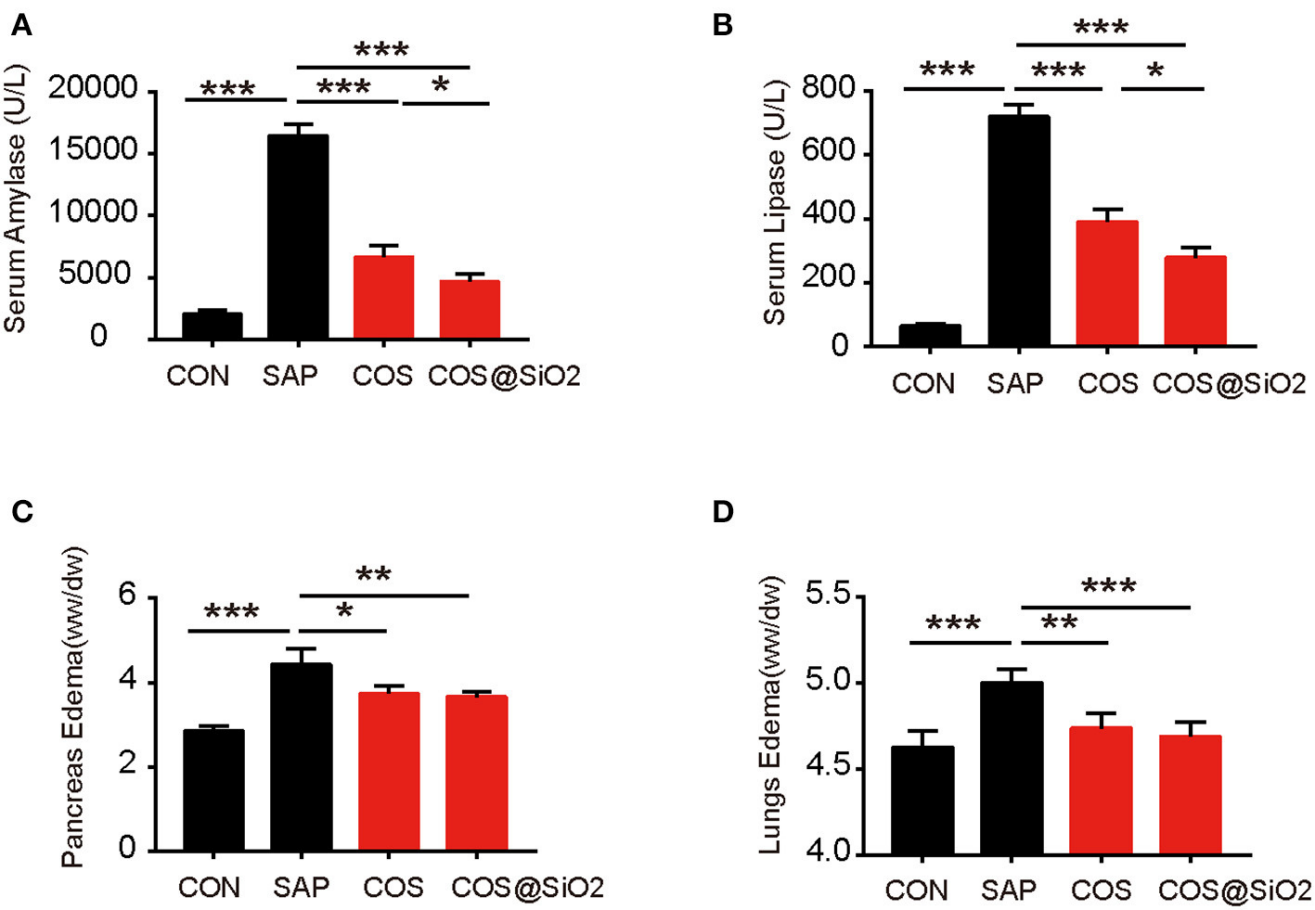
Porous COS@SiO_2_ nanocomposites alleviate pancreatic and lung injury in mice with caerulein-induced acute pancreatitis (AP). COS@SiO2 reduced **(A)** the level of serum amylase and **(B)** the level of serum lipase and improved **(C)** pancreatic edema [wet–dry (W/D) ratio] and **(D)** lung edema (W/D ratio). ww, wet weight; dw, dry weight. Pancreatic and lung tissues were weighed and the W/D ratio was calculated. The data are provided as the mean ± SEM (*n* = 6 per group). *p < 0.05; **p < 0.01; ***p < 0.001.

In the SAP group, we also found out that the specific indices of AP (including serum amylase and serum lipase) were significantly increased compared with the CON group. The same indices in the COS and COS@SiO_2_ groups were obviously lower than in the SAP group and were significantly different (*p* < 0.001; Figures 3A,B). In addition, we assayed the pancreatic and lung wet-to-dry weight ratio in each group. This showed the same trend as the serum amylase and lipase (Figures 3C,D). These data indicate that COS@SiO_2_ can reduce pancreatic and pulmonary edema.

To verify the effectiveness of the porous COS@SiO_2_ nanocomposites, we used another well-known SAP model induced by L-arginine injection. In the same way as in the model of caerulein-induced SAP, porous COS@SiO_2_ nanocomposites also attenuated injury to the pancreas and lung induced by L-arginine in a dose-dependent manner (Supplementary Figure 3). These data show that COS@SiO_2_ is effective in both caerulein-induced and L-arginine-induced AP.

All in all, these results proved that COS@SiO_2_ ameliorated not only pancreatic injury but also lung injury.

### Porous COS@SiO_2_ Nanocomposites Reduced Inflammation in Mice With Caerulein-Induced AP

**Figure 4:**
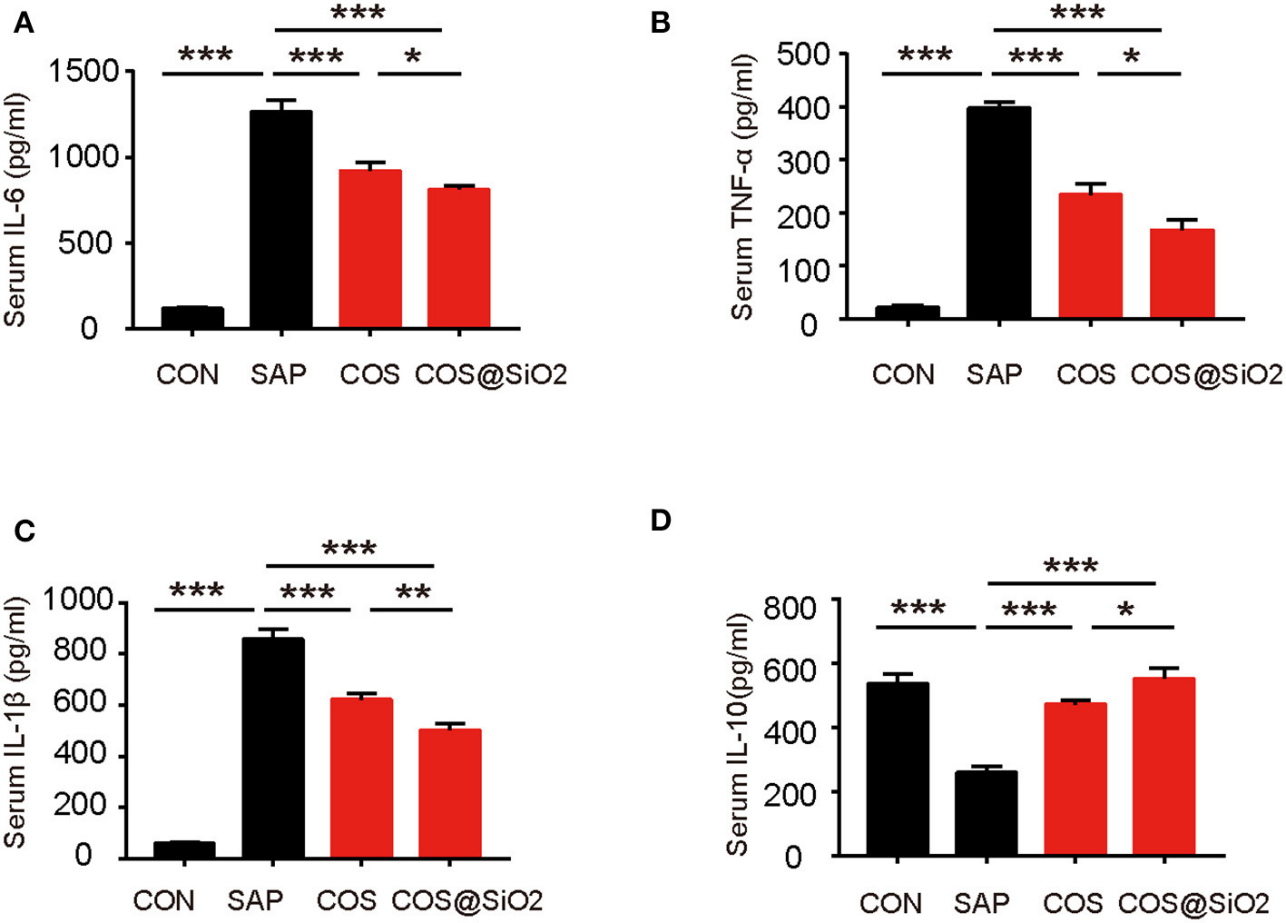
Porous COS@SiO_2_ nanocomposites reduced systemic inflammation. COS@SiO2 decreased **(A)** the serum level of interleukin (IL)-6, **(B)** the serum level of tumor necrosis factor-α (TNF-α), and **(C)** the serum level of IL-1β and increased **(D)** the serum level of IL-10. The data are provided as the mean ± SEM (*n* = 6 per group). *p < 0.05; **p < 0.01; ***p < 0.001.

Compared with the CON group, the serum level of proinflammatory cytokines, including IL-6, IL-1β, and TNF-α, was significantly higher in the SAP group, while the serum level of IL-10, a type of anti-inflammatory cytokine, was lower (*p* < 0.001). We analyzed the same indices in COS-treated groups. ELISA showed that the serum level of proinflammatory cytokines (IL-6, IL-1β, and TNF-α) decreased and that the level of anti-inflammatory cytokine (IL-10) increased significantly (*p* < 0.001; Figures 4A–D). These results suggest that COS@SiO_2_ could alleviate AP-mediated systemic inflammation.

### Porous COS@SiO_2_ Nanocomposites Inhibited Oxidative Stress in Mice With Caerulein-Induced AP

**Figure 5:**
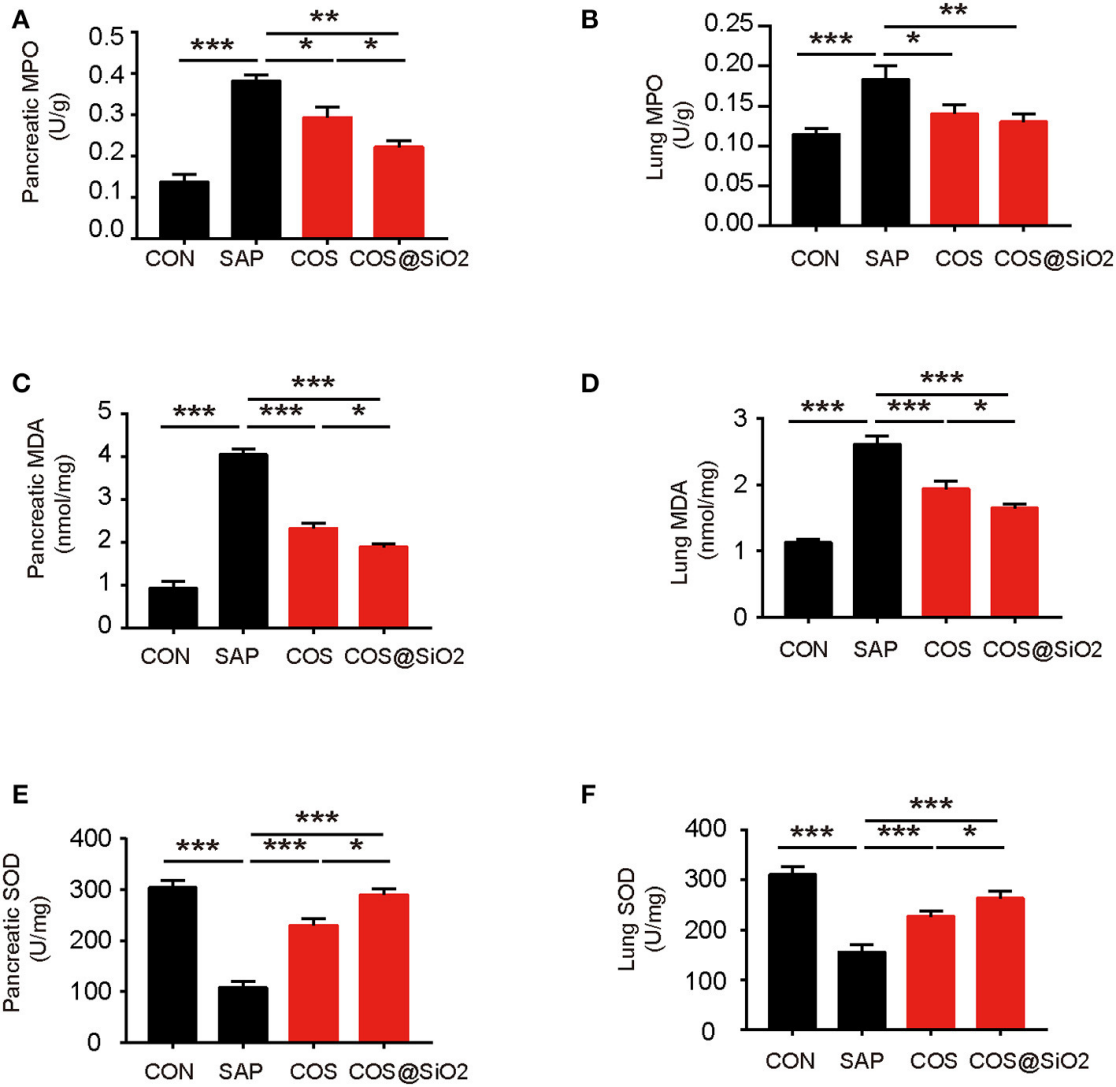
Porous COS@SiO_2_ nanocomposites reduced oxidative stress in both the pancreas and lung. **(A)** The level of pancreatic myeloperoxidase (MPO) in each group. **(B)** The level of lung MPO in each group. **(C)** The level of pancreatic malondialdehyde (MDA) in each group. **(D)** The level of lung MDA in each group. **(E)** The level of pancreatic superoxide dismutase (SOD) in each group. **(F)** The level of lung SOD in each group. The data are provided as the mean ± SEM (*n* = 6 per group). *p < 0.05; **p < 0.01; ***p < 0.001.

Compared with the CON group, the level of oxidation-related indices, including MPO and MDA, in pancreatic and lung tissues was obviously increased in the SAP group, while the antioxidation related index (SOD) was decreased (*p* < 0.001). We also assayed the same indices in the COS- and COS@SiO_2_-treated groups. The results showed that the levels of MPO and MDA were lower and the level of SOD was higher than in the SAP group (*p* < 0.05). Furthermore, we found that the COS@SiO_2_ group showed lower MPO and MDA levels and a higher level of SOD (Figure 5). These results indicate that COS@SiO_2_ could inhibit oxidative stress more than using COS alone.

### Porous COS@SiO_2_ Nanocomposites Suppressed NLRP3-Mediated Inflammasome and NF-κB Activation by Regulating the Nrf2 Signaling Pathway in Mice With Caerulein-Induced AP

**Figure 6:**
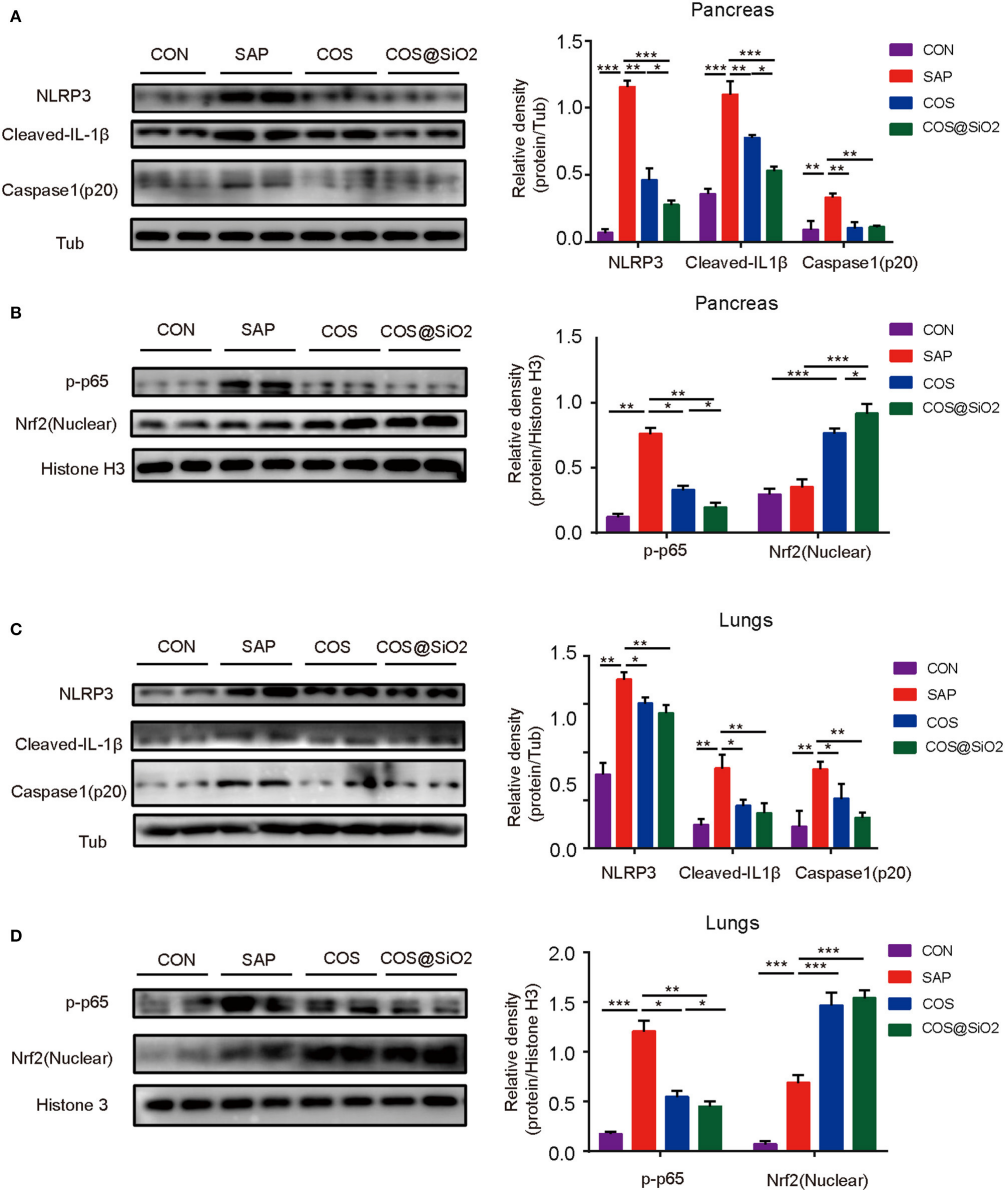
Porous COS@SiO_2_ nanocomposites suppressed the NOD-like receptor protein 3 (NLRP3)-mediated inflammasome and nuclear factor-kappa B (NF-κB) activation by regulating the nuclear factor E2-related factor 2 (Nrf2) signaling pathway in mice with caerulein-induced AP. The protein levels of **(A)** the NLRP3 inflammasome and β-tubulin and **(B)** Nrf2, phospho-NF-κB (p-NF-κB), and histone in pancreas were analyzed by western blotting. Also, the protein levels of **(C)** the NLRP3 inflammasome and β-tubulin and **(D)** Nrf2, p-NF-κB, and histone in lungs were also analyzed by western blotting. The data are provided as the mean ± SEM (*n* = 6 per group). *p < 0.05; **p < 0.01; ***p < 0.001.

The components of the NLRP3 inflammasome/IL-1β secretion axis and NF-κB were significantly upregulated in the pancreas and lung of the SAP group compared with the CON group (*p* < 0.01). These effects could be inhibited by the COS and COS@SiO2 treatment. Furthermore, we found that the expression of Nrf2 protein was markedly increased in the pancreas and lung of the COS@SiO_2_-treated group (Figure 6). These results indicate that COS@SiO_2_ may upregulate Nrf2 to reduce the NLRP3 inflammasome and NF-κB activation.

**Figure 7:**
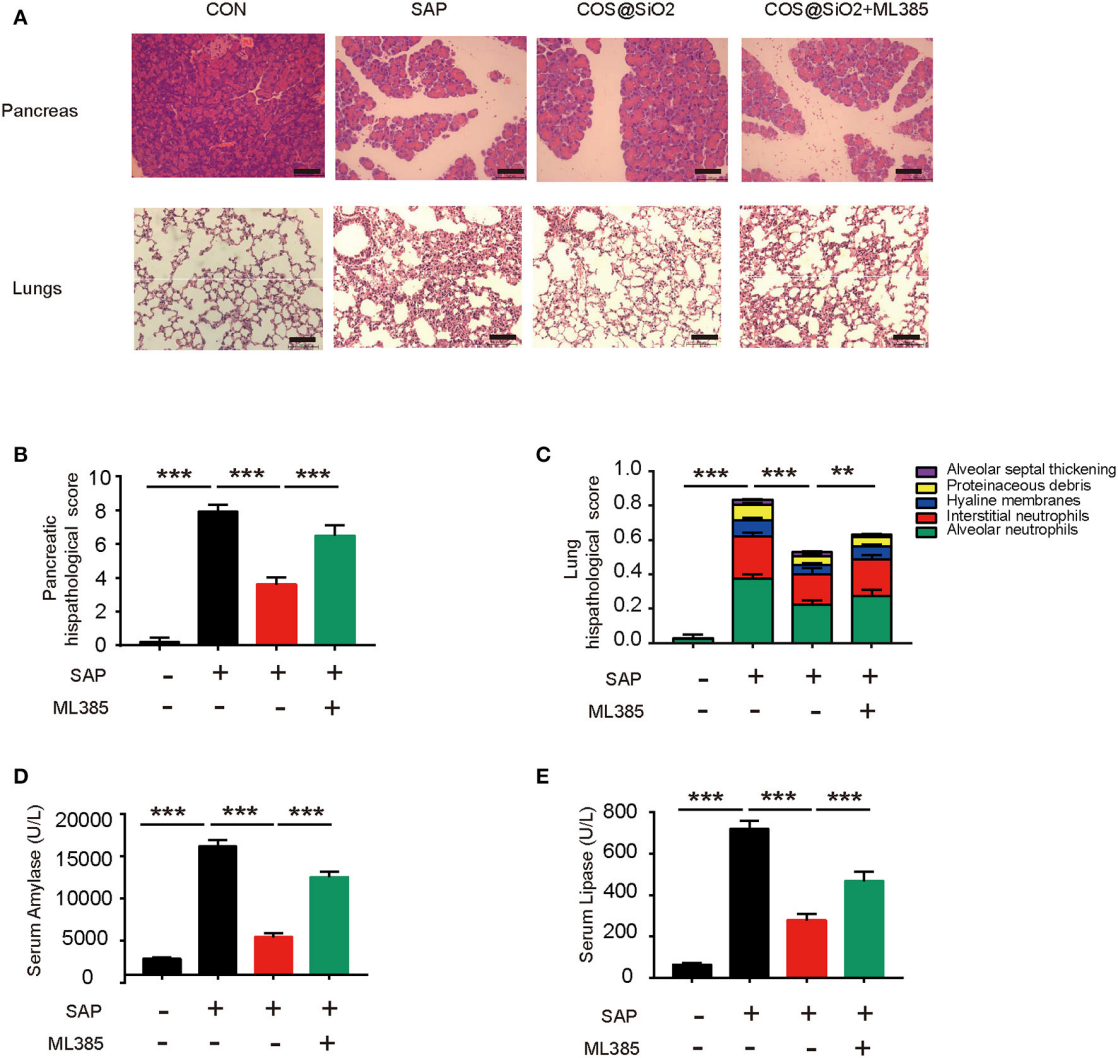
The protective effect of porous COS@SiO_2_ nanocomposites in mice with caerulein-induced AP could be reversed by nuclear factor E2-related factor 2 (Nrf2) inhibition. ML385, a type of Nrf2 inhibitor, was used to determine whether it could reduce the protective effect of porous COS@SiO2 nanocomposites. **(A)** Pancreatic and lung samples from each group of mice were stained with H&E. Representative images of pancreas and lung are shown. Original magnification ×100. **(B)** Pancreatic histopathology scores were evaluated by Schmidt criteria for pancreatic tissue by edema, hemorrhage, necrosis, and inflammatory infiltration. **(C)** Lung histopathology scores were evaluated by alveolar neutrophils, interstitial neutrophils, hyaline membranes, proteinaceous debris, and alveolar septal thickening. **(D)** The level of serum amylase in each group mice. **(E)** The level of serum lipase in each group mice. The data are provided as the mean ± SEM (*n* = 6 per group). **p < 0.01; ***p < 0.001.

ML385, a type of Nrf2 inhibitor, was used to inhibit Nrf2 signaling in mice with caerulein-induced AP. Compared with the COS@SiO_2_ nanocomposite treatment group, the pathological injury to the pancreas and lung in the intervention group with ML385 was more serious, the pathological score was statistically different (*p* < 0.01), and the serum amylase and lipase levels were significantly higher (*p* < 0.001; Figure 7). This suggests that ML385 could reverse the protective effect of COS@SiO_2_. In other words, COS@SiO_2_ played a protective role in AP through the Nrf2 signaling pathway.

## Discussion

In this study, we determined the protective effects of porous COS@SiO_2_ nanocomposites on caerulein-induced SAP in mice. We demonstrated that COS@SiO_2_ could significantly reduce pancreatic and lung pathological damage in SAP and was more effective than COSs alone. Further investigations showed that COS@SiO_2_ inhibited the systemic and local (pancreas and lung) inflammation and oxidative stress by activating the Nrf2 signaling pathway.

COSs are oligosaccharides with a degree of polymerization between 2 and 20 that are obtained by degradation of chitosan. They are mixtures of β-1,4-linked D-glucosamine residues (Muanprasat and Chatsudthipong, 2017). Compared with high molecular weight chitosan, COSs have better water solubility; are more easily absorbed, transformed, and utilized *in vivo*; have higher reactivity; and have more important biological functions (Yoon et al., 2008). COSs are well-known for their antioxidative stress ability. Because of their biocompatibility, biodegradability, non-toxicity, and absorption properties, COSs have been recommended as agents that are superior to other antioxidants (Nidheesh et al., 2016; Naveed et al., 2019). As a prebiotic with full solubility, COSs have been linked to various health-promoting potentials, including regulating glucose and lipid metabolism, promoting calcium absorption, and maintaining the integrity of the gut barrier (Jung et al., 2006; Bai et al., 2018). Moreover, COSs have been shown to reduce the effects of many diseases, such as LPS-induced ALI (Liu et al., 2018) and fatty liver disease (du Plessis et al., 2019) induced by a high fat diet, and so on.

Porous silica nanoparticles form a flocculent amorphous white powder that is non-toxic, odorless, and has good biocompatibility, water solubility, easy modification, thermal stability, high specific surface area, and a regular and uniform pore structure (Al-Sagheer and Muslim, 2010). In contrast, owing to the SiO_2_ coating structure, porous COS@SiO_2_ nanocomposites have obvious advantages in terms of economy and stability (room temperature storage).

In addition, our study proves that, in a weak alkaline environment as occurs in AP, the release rate of COSs from porous COS@SiO_2_ nanocomposites is higher and the release speed of COSs is faster. This feature allows COSs to play a biological role at smaller doses, thereby reducing side effects.

All in all, porous silica nanoparticles give COSs the advantage of pH-controlled, sustained release to maintain a high concentration, overcome the shortcomings of easy degradation of COSs, and also give COSs the advantage of accumulating in the pancreas.

AP is an inflammatory reactive disease commonly found in the clinic. It has a rapid onset and develops rapidly. ALI in SAP is a common and early complication, and also one of the most important causes of death from SAP (Forsmark et al., 2016).

The levels of ROS increase significantly from the injured pancreatic acinar cells and activated immune cells in AP (Chvanov et al., 2005). ROS are a type of oxygen-containing active substance with high reactivity. Pancreatic injury is highly correlated with oxidative stress (Steinbrenner and Sies, 2009). On one hand, ROS directly cause lung cell damage and control intercellular signal transduction. On the other hand, ROS are also involved in polymorphonuclear activation, cytokine production, and disturbance of the endothelial barrier and microcirculation (Guice et al., 1989).

We used COS@SiO_2_ to pre-treat mice with SAP and found that the treated groups showed reduced damage to the pancreas and lungs and decreased systemic and local inflammation compared with the untreated AP group. In addition, we also proved the anti-oxidative stress ability of COS@SiO_2._

During AP, the increased levels of ROS cause NF-κB, one of the most important proinflammatory factors, to be activated. NF-κB moves from the cytoplasm into the nucleus and this results in the release of downstream inflammatory cytokines, including TNF-α, IL-6, IL-1β, and so on, activating NF-κB (Huang et al., 2013; Zhang et al., 2018). As previous studies have shown, these inflammatory cytokines are closely related to the development of AP (Wirth and Baltimore, 1988). In addition, ROS activate the NLRP3 inflammasome. The NLRP3 inflammasome consists of NLRP3, apoptosis-associated speck-like protein containing CARD (ASC), and cysteinyl aspartate-specific proteinase-1 (caspase-1). The NLRP3 inflammasome forms a molecular platform that activates caspase-1, which can catalyze the proteolytic process and secrete mature IL-1β, which is a powerful proinflammatory factor that can trigger a series of inflammatory reactions (Loukovaara et al., 2017).

In our study, we have shown that, in caerulein-induced AP, the level of oxidative stress-related indices (MDA, MPO) in the pancreas and lung, the expression of the NLRP3 inflammasome, and the level of NF-κB protein in the pancreas and lungs as well as the levels of inflammatory cytokines (TNF-α, IL-6, IL-1β) in the serum and pancreatic tissues increase obviously, which could reflect the activation of oxidative stress and inflammation. As a result, we surmise that oxidative stress is the key to the pathogenesis of AP, which means that the increased ROS levels lead to an inflammatory cascade effect. In other words, COS@SiO_2_ may be an effective treatment for AP because of its ability to inhibit oxidative stress. Furthermore, we explored the antioxidative stress mechanism of COS@SiO_2_.

Nrf2 is an important transcription factor in the leucine zipper family that regulates oxidative stress, and its specific receptor is Kelch-like ECH-related protein 1 (Keap1). Normally, Keap1 and Nrf2 are present in the cytoplasm in the form of compounds (Yamamoto et al., 2018; Qin et al., 2019). Under oxidative stress, Keap1 dissociates from Nrf2, and then Nrf2 is translocated into the nucleus and integrated with antioxidant response elements and regulated antioxidant proteins such as SOD, which could protect the body from ROS (Motohashi and Yamamoto, 2004).

In our study, western blot analysis showed that, combined with the treatment of COS@SiO_2_, the expression of Nrf2 protein was increased and the expression of NF-κB and the NLRP3 inflammasome protein was decreased in both lung and pancreatic tissue, which indicated that COS@SiO_2_ might inhibit oxidative stress by upregulating the Nrf2 signaling pathway.

To prove whether Nrf2 plays an important role, we also used ML385, a type of Nrf2 inhibitor, in COS@SiO_2_-treated mice with AP. The results showed that the protective effects of porous COS@SiO_2_ nanocomposites was reversed by inhibiting the Nrf2 signal.

## Conclusion

In conclusion, we have demonstrated that porous COS@SiO_2_ nanocomposites activate the Nrf2 signaling pathway to inhibit oxidative stress and reduce the production of NF-κB and NLRP3 and the release of inflammatory factors, thus blocking the systemic inflammatory response and ultimately ameliorating SAP and the associated lung injury.

## Data Availability Statement

The original contributions presented in the study are included in the article/Supplementary Material, further inquiries can be directed to the corresponding author/s.

## Ethics Statement

All experiments involving animals were conducted under the principle for replacement, refinement, reduction (the 3Rs) and according to the legislation on the protection of animals and were approved by the Animal Ethics Committee of Shanghai Jiaotong University School of Medicine (SYXK 2013-0050, Shanghai, China).

## Author Contributions

GD and QM designed the research plan. QM and ZH performed the research and generated the draft of the manuscript. YY, JH, and YF assisted in the animal experiments, and CL interpreted the results. XW and YZ edited and revised the manuscript. All authors read and approved the final version of the manuscript.

## Conflict of Interest

The authors declare that the research was conducted in the absence of any commercial or financial relationships that could be construed as a potential conflict of interest.

## Footnotes

^1^dried by using blotting paper.

## Supplementary Material

Supplementary Figure 1 A description of porous COS@SiO2 nanocomposites attenuating acute pancreatitis and associated lung injury. Porous COS@SiO2 nanocomposites ameliorate severe acute pancreatitis and associated lung injury by regulating the nuclear factor E2-related factor 2 (Nrf2) signaling pathway to inhibit oxidative stress and reduce nuclear factor-kappa B (NF-κB) and the NOD-like receptor protein 3 (NLRP3) inflammasome.

Supplementary Figure 2 Porous COS@SiO2 nanocomposites were safe in the pancreas and lungs. Pancreatic and lung samples from each group of mice were stained with H&E. Representative images of pancreas and lung are shown. Original magnification ×100.

Supplementary Figure 3 Porous COS@SiO2 nanocomposites also ameliorated pathological injury to the pancreas and lung in mice with L-arginine-induced severe acute pancreatitis. Pancreatic and lung samsples from each group of mice were stained with H&E. **(A)** Representative images of the pancreas and lung are shown. Original magnification ×100. **(B)** Pancreatic and **(C)** lung histopathology scores were evaluated as before. The data are provided as the mean ± SEM (n = 6 per group). ^*^*p* < *0.05;* ^**^*p* < *0.01;* ^***^*p* < 0.001.
