# Supplementary figures and images for: Porous COS@SiO2 Nanocomposites Ameliorate Severe Acute Pancreatitis and Associated Lung Injury by Regulating the Nrf2 Signaling Pathway in Mice
Source: Front Chem. 2020 Oct 7;8:720. doi: 10.3389/fchem.2020.00720 (PMC7579426; doi:10.3389/fchem.2020.00720)

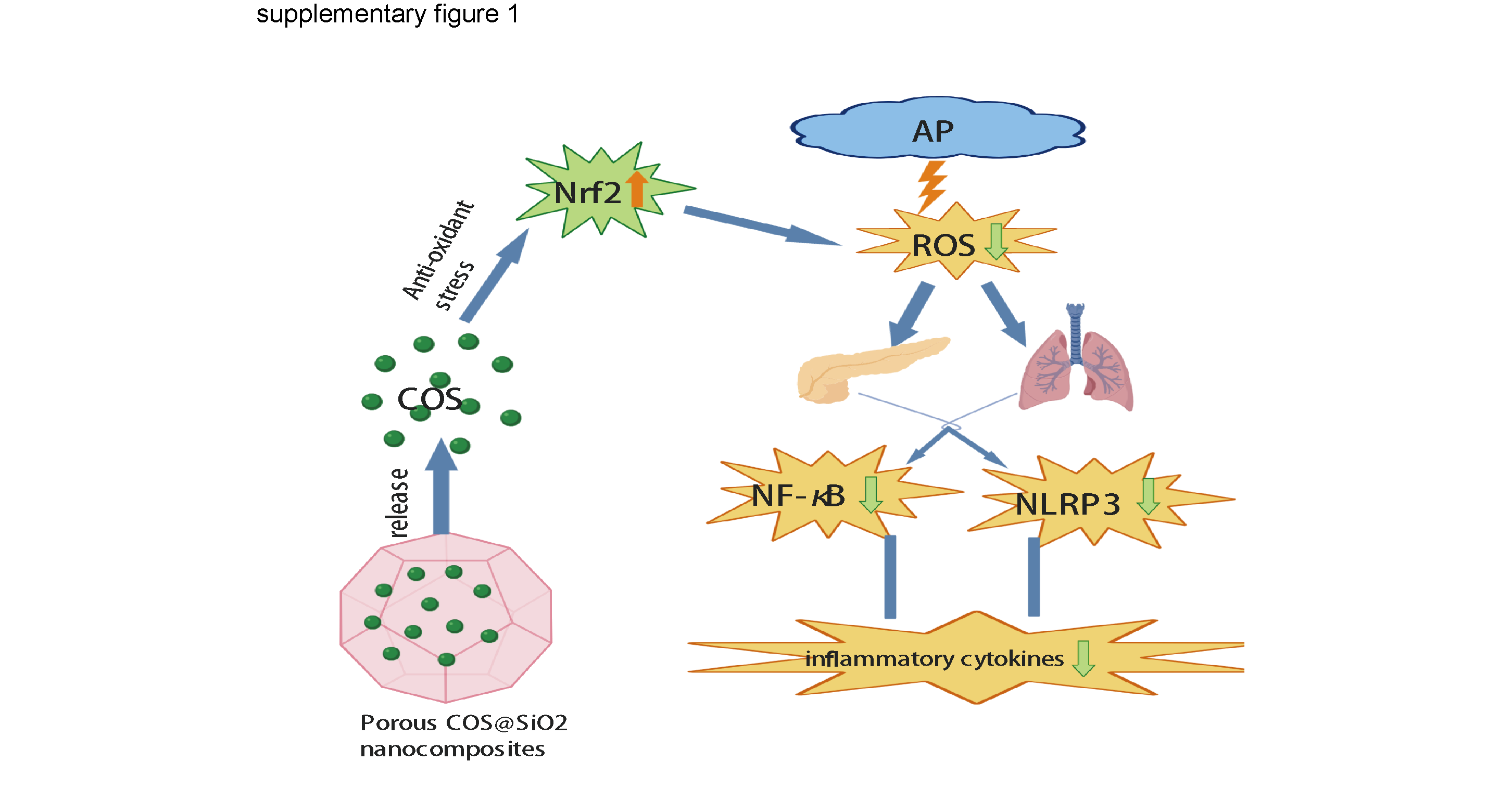

Supplement: Supplementary Figure 1 — A description of porous COS@SiO2 nanocomposites attenuating acute pancreatitis and associated lung injury. Porous COS@SiO2 nanocomposites ameliorate severe acute pancreatitis and associated lung injury by regulating the nuclear factor E2-related factor 2 (Nrf2) signaling pathway to inhibit oxidative stress and reduce nuclear factor-kappa B (NF-κB) and the NOD-like receptor protein 3 (NLRP3) inflammasome. [file Image_1.TIF]

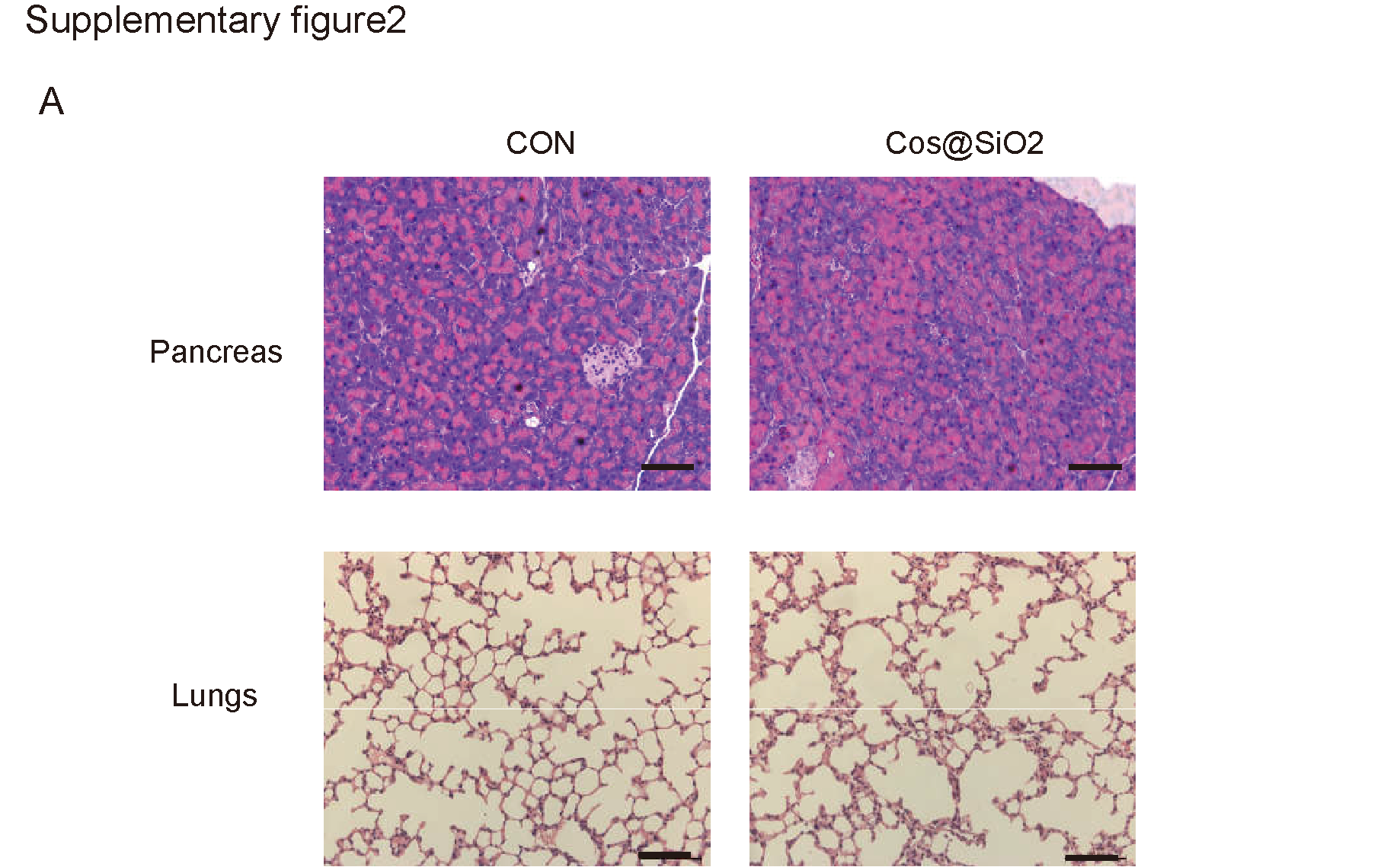

Supplement: Supplementary Figure 2 — Porous COS@SiO2 nanocomposites were safe in the pancreas and lungs. Pancreatic and lung samples from each group of mice were stained with H&E. Representative images of pancreas and lung are shown. Original magnification ×100. [file Image_2.TIF]

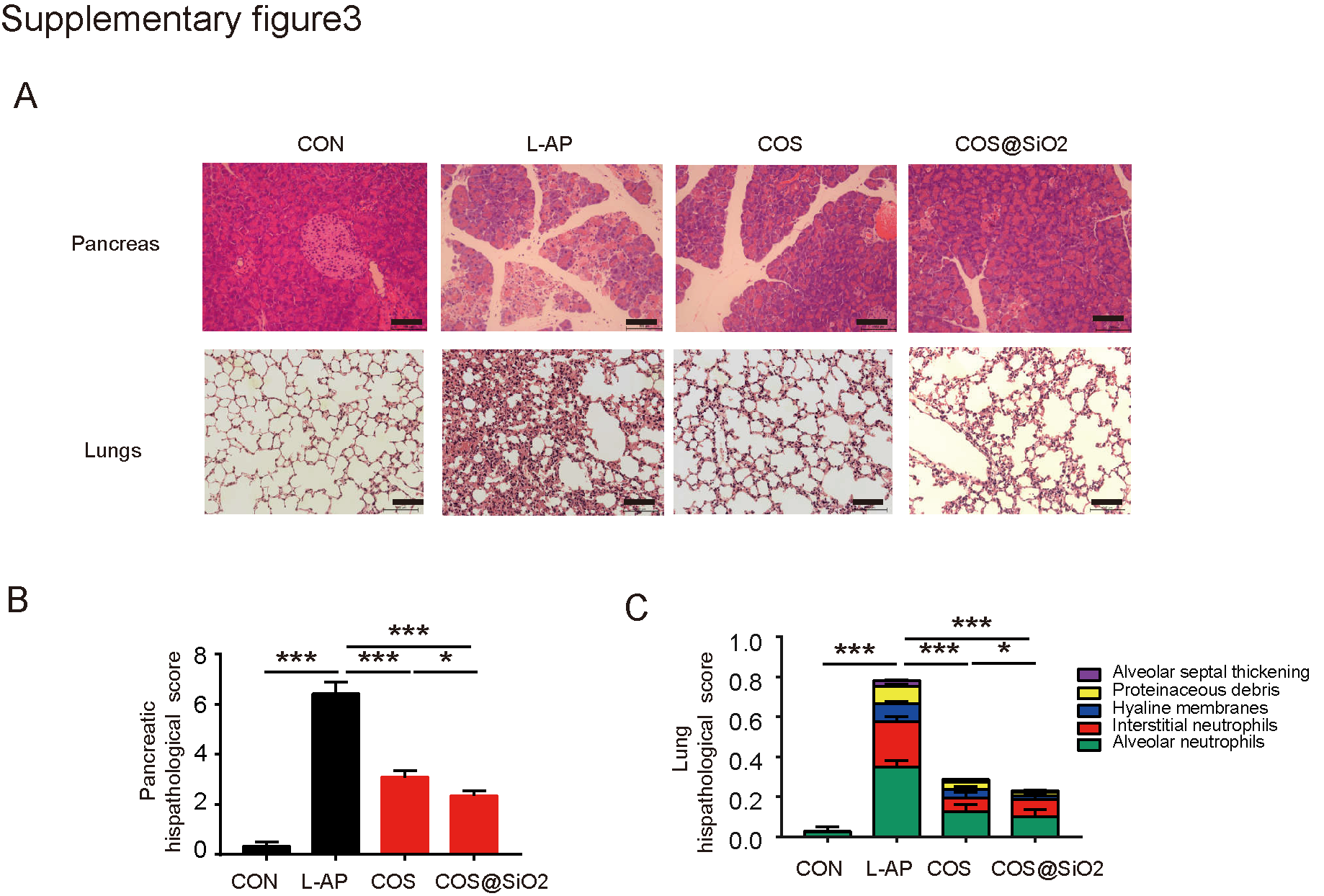

Supplement: Supplementary Figure 3 — Porous COS@SiO2 nanocomposites also ameliorated pathological injury to the pancreas and lung in mice with L-arginine-induced severe acute pancreatitis. Pancreatic and lung samsples from each group of mice were stained with H&E. (A) Representative images of the pancreas and lung are shown. Original magnification ×100. (B) Pancreatic and (C) lung histopathology scores were evaluated as before. The data are provided as the mean ± SEM (n = 6 per group). *p < 0.05; **p < 0.01; ***p < 0.001. [file Image_3.TIF]
